# Crizanlizumab for retinal vasculopathy with cerebral leukoencephalopathy in a phase II clinical study

**DOI:** 10.1172/JCI180916

**Published:** 2024-05-07

**Authors:** Wilson X. Wang, Dan Spiegelman, P. Kumar Rao, Andria L. Ford, Rajendra S. Apte

**Affiliations:** 1 John F. Hardesty, MD Department of Ophthalmology and Visual Sciences, and; 2 Department of Neurology, Washington University School of Medicine, St. Louis, Missouri, USA.; 3 Department of Medicine, Washington University, St. Louis, Missouri, USA.

**Keywords:** Clinical trials, Ophthalmology, Retinopathy

## Abstract

**Background:**

Retinal vasculopathy with cerebral leukoencephalopathy and systemic manifestations (RVCL-S) is a rare, autosomal dominant, universally fatal disease without effective treatment options. This study explores the safety and preliminary efficacy of crizanlizumab, a humanized monoclonal antibody against P-selectin approved for the prevention of sickle cell crises, in slowing retinal nonperfusion and preserving vision in patients with RVCL-S.

**METHODS:**

Eleven patients with RVCL-S with confirmed exonuclease 3 prime repair exonuclease 1 (*TREX1*) mutations received monthly crizanlizumab infusions over 2 years. The study measured the nonperfusion index within 3 retinal zones and the total retina with fluorescein angiography, visual acuity, intraocular pressure (IOP), and optical coherence tomography central subfield thickness (CST) at baseline, 1 year, and 2 years. A mixed repeated-measures analysis was performed to assess the progression rates and changes from baseline.

**RESULTS:**

Eleven participants received crizanlizumab infusions. All of the participants tolerated crizanlizumab well, with 8 of 11 (72.7%) reporting mild adverse effects such as nausea, fatigue, and gastrointestinal symptoms. The change in total retinal nonperfusion was 7.22% [4.47, 9.97] in year 1 and –0.69% [–4.06, 2.68] in year 2 (*P* < 0.001). In the mid periphery, the change in nonperfusion was 10.6% [5.1, 16.1] in year 1 and –0.68% [–3.98, 5.35] in year 2 (*P* < 0.01), demonstrating a reduction in progression of nonperfusion in the second year of treatment. Visual acuity, IOP, and CST remained stable.

**CONCLUSION:**

Crizanlizumab has an acceptable safety profile. These results show promising potential for examining crizanlizumab in larger studies of RVCL-S and similar small-vessel diseases and for using the retina as a biomarker for systemic disease.

**Trial registration:**

ClinicalTrials.gov NCT04611880.

**FUNDING:**

The Clayco Foundation; DeNardo Education and Research Foundation Grant; Jeffrey T. Fort Innovation Fund; Siteman Retina Research Fund; unrestricted grant from Research to Prevent Blindness Inc.; National Heart,Lung, and Blood Institute (NHLBI), NIH (R01HL129241); National Institute of Neurological Disorders and Stroke (NINDS), NIH (RF1NS116565).

## Abstract 2

Patients tolerated crizanlizumab infusions for retinal vasculopathy with cerebral leukoencephalopathy and systemic manifestations and showed reduced progression of nonperfusion in the second year of treatment.

## Introduction

Retinal vasculopathy with cerebral leukoencephalopathy and systemic manifestations (RVCL-S) is a rare, autosomal dominant disorder characterized by a spectrum of genetic and pathophysiological features (1). Identified in fewer than 25–40 families worldwide since its first report in the 1980s, RVCL-S is caused by C-terminus mutations in the exonuclease 3 prime repair exonuclease 1 (*TREX1*) gene (2). This condition manifests typically in the third or fourth decade of life, leading to vision loss, mini-strokes, nephropathy, and ultimately cognitive decline and premature death around the age of 50. The disease’s complete penetrance, with blindness and early-onset dementia as primary morbidity factors, underscores the urgent need for effective treatments (3). The disease primarily manifests as a microvasculopathy affecting the eyes and brain. Technological advancements in imaging modalities have allowed for a more detailed understanding of the extent of endothelial involvement of retinal vessels and the structural and functional effect of RVCL-S on the retina (4). Despite these substantial advances in our understanding of the effects of RVCL-S on the neurosensory retina, therapeutic options remain largely limited to treating complications of ischemic retinal vasculopathy, with anti-VEGF therapy and laser panretinal photocoagulation (PRP) for proliferative retinopathy and neovascular glaucoma (NVG) or surgical interventions for vitreous hemorrhage and uncontrolled intraocular pressure (IOP) secondary to NVG. Currently, RVCL-S lacks systemic or targeted therapies that are disease modifying (5).

Although designing effective therapeutic strategies to manage RVCL-S is challenging due to its rarity, previous attempts at disease-modifying treatments have been guided by the association of *TREX1* with autoimmune and autoinflammatory diseases (6). Corticosteroids such as prednisone have been studied as potential treatment options, however they do not change the long-term disease trajectory and have considerable side effects when used chronically (7). Immunosuppressants like cyclophosphamide, methotrexate, and azathioprine have seen similar limited success because of side effects and lack of efficacy. A phase I study of aclarubicin aimed at correcting the glycan defects due to dysregulation of oligosaccharyltransferase — a prevailing theory in RVCL-S pathophysiology — was an important step in the conception and undertaking of clinical trials for patients with this rare disease, despite its discontinuation due to lack of benefit in 4 patients. Nonetheless, this pioneering study paved the way for future clinical trials in RVCL-S and other rare diseases, highlighting the importance of continuous research for viable treatment options (5).

Since then, a growing body of research regarding the pathophysiology of RVCL-S suggests that endothelial dysfunction may lead to microvascular occlusion and ischemia, prompting a renewed interest in therapies that target these pathways and have an acceptable safety profile. Crizanlizumab, a P-selectin antagonist initially developed for the prevention of sickle cell pain crises, has emerged as a promising candidate (8). This humanized monoclonal P-selectin antibody prevents leukocyte adhesion to P-selectin on activated vascular endothelium, potentially reducing microvascular occlusions, and leading to fewer sickle-induced pain crises (9). Crizanlizumab’s effectiveness in limiting sickle cell pain crises may indicate a similar potential of mitigating microvascular occlusive disease and the subsequent ischemia that contributes to microvasculopathies like RVCL-S. By preventing leukocyte-endothelium interactions, crizanlizumab could decrease the incidence of ischemic lesions in the brain and slow the progression of retinal nonperfusion, presumably driven by microvascular occlusions, thereby preserving vision in patients with RVCL-S.

This clinical study aimed to assess the safety and preliminary efficacy of crizanlizumab in patients with RVCL-S, marking an important step in the understanding and treatment options of this rare and severe disease. We investigated the rate of progression of retinal nonperfusion and other key indicators including changes in visual acuity, IOP, and retinal thickness. Our research into crizanlizumab’s utility as a therapeutic for RVCL-S is intended not only to advance management strategies for the retinal complications of the disease but also to enhance our understanding of similar vasculopathies affecting the retina.

## Results

### Baseline characteristics

**Figure 1:**
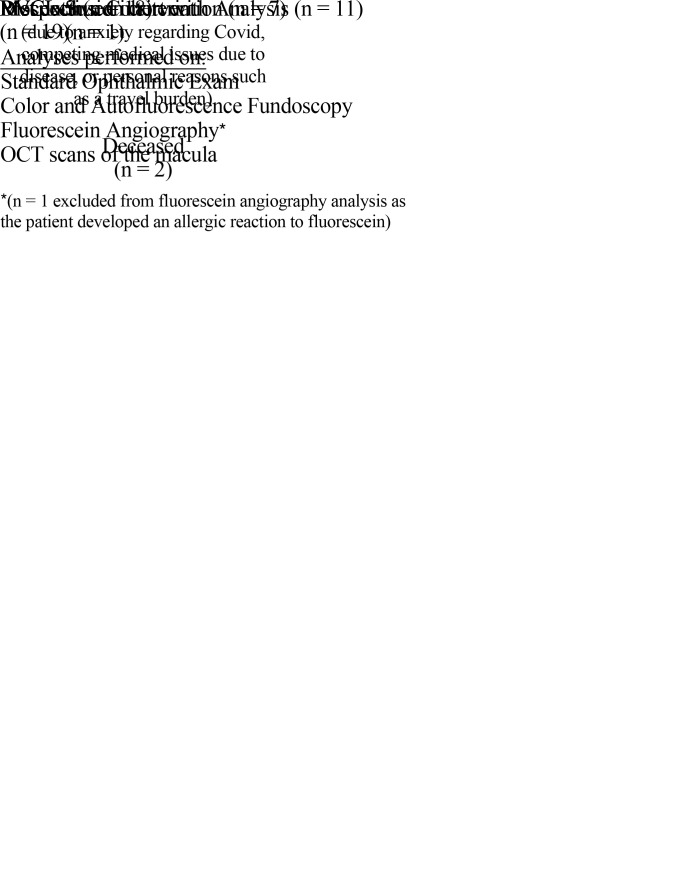
CONSORT diagram displaying the flow of RVCL-S patients through the 2-year protocol from initial assessment for eligibility to protocol completion with corresponding analyses performed.

From January to December 2021, a total of 19 patients with confirmed *TREX1* mutations were screened. Eighteen of these patients met the enrollment criteria and were included in the study (Figure 1). Of the enrolled cohort, 7 patients discontinued the treatment within 2 to 8 months. One patient developed health problems related to the progression of RVCL-S, was placed on comfort care, and died from natural disease progression unrelated to the study medication. Another participant withdrew because of the travel burden and later died as a result of disease complications from natural disease progression. The other 5 patients withdrew because of a combination of health challenges, travel constraints, and personal factors. The study continued with 11 participants whose baseline characteristics are summarized in Table 1. The mean age of these participants was 45.7 years, ranging from 36–57 years, with a female predominance (72.7%). During the trial, only 3 patients required anti-VEGF injections, as clinically indicated, and none underwent laser photocoagulation. Baseline assessments recorded a median visual acuity of 20/22 and a central subfield thickness (CST) of 265.5 μm. Patients who completed the study and those who withdrew had a difference in the average time since diagnosis of 1.6 years and 5 years, respectively.

### Safety and tolerability

In this study, 11 patients completed the regimen of crizanlizumab infusions. The treatment was well tolerated, with no instances of grade 3–4 adverse effects directly attributable to crizanlizumab. Furthermore, there were no treatment-related fatalities. Adverse effects, predominantly of mild-to-moderate severity (grades 1–2), included constitutional symptoms (48%) and gastrointestinal disturbances (35%), as detailed in Table 2. Among constitutional symptoms, nausea (54.5%) and fatigue (36.4%) were the most common, followed by vomiting, general weakness, and headache. Gastrointestinal side effects encompassed diarrhea, loose stool, with or without incontinence, and abdominal cramps. The clinical monitoring team observed that crizanlizumab infusions were generally well tolerated, with minimal effect on the participant’s wellbeing. There were no unanticipated toxicities, and all adverse events were managed effectively in accordance with established clinical protocols.

### Standard ophthalmic examination

#### Changes in BCVA and IOP.

**Figure 2:**
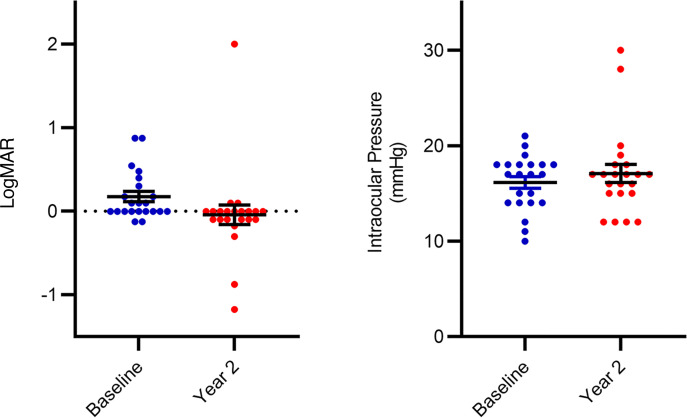
logMAR and IOP values. logMAR and IOP (mmHg) values for 22 RVCL-S patient eyes, with the mean ± SEM at baseline (blue) and year 2 (red).

The average logMAR was 0.176 (Snellen equivalent between 20/25 and 20/30) and –0.042 (Snellen equivalent between 20/15 and 20/20) at baseline and year 2, respectively (*P* = 0.10). Individual cases of substantial vision loss underscore the variability of the underlying condition as well as patient response, as some individuals experienced considerable vision loss over the 2 years, worsening from 20/70 to 2/300 or 20/150 for counting fingers over the course of treatment due to natural disease progression and disease-related ophthalmic complications. IOP was 16.1 mmHg [14.9, 17.4] and 17.1 mmHg [15.1, 19.1] at baseline and year 2, respectively (*P* = 0.20) (Figure 2). Both eyes of 1 patient had elevated IOP during the study, however there was no evidence of neovascular glaucoma, and subsequent IOP measurements were normal for that patient.

#### Changes in CST.

**Figure 3:**
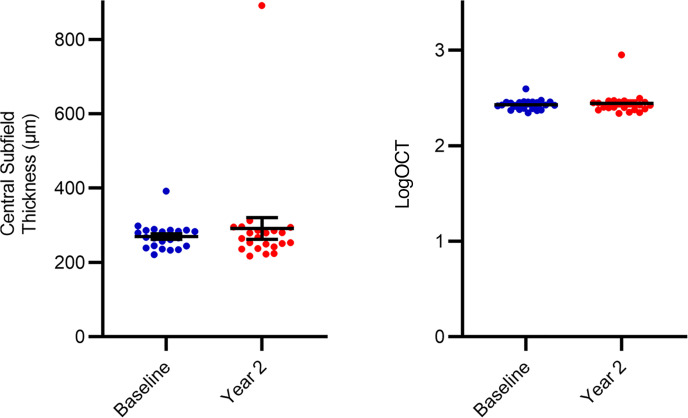
OCT CST and logOCT values. OCT CST and logOCT values for 22 RVCL-S patient eyes with mean ± SEM at baseline (blue) and year 2 (red).

Mean CST was 270 μm (95% CI [254, 285]) and 291 μm (95% CI [231, 352]) at baseline and year 2, respectively (*P* = 0.35). Mean log optical coherence tomography (logOCT) was 2.43 (95% CI [2.40, 2.45]) and 2.44 (95% CI [2.39, 2.50]) at baseline and year 2, respectively (*P* = 0.42) (Figure 3). One notable instance of elevated CST included a patient with cystoid macular edema, underscoring the potential for macular complications associated with RVCL-S vasculopathy. The Mann-Whitney *U* test *P* values comparing baseline and year 2 with the normative database for CST were *P* = 0.19 and *P* = 0.12, respectively.

### Efficacy outcomes

#### Nonperfusion index and rates of change of nonperfusion area.

**Figure 4:**
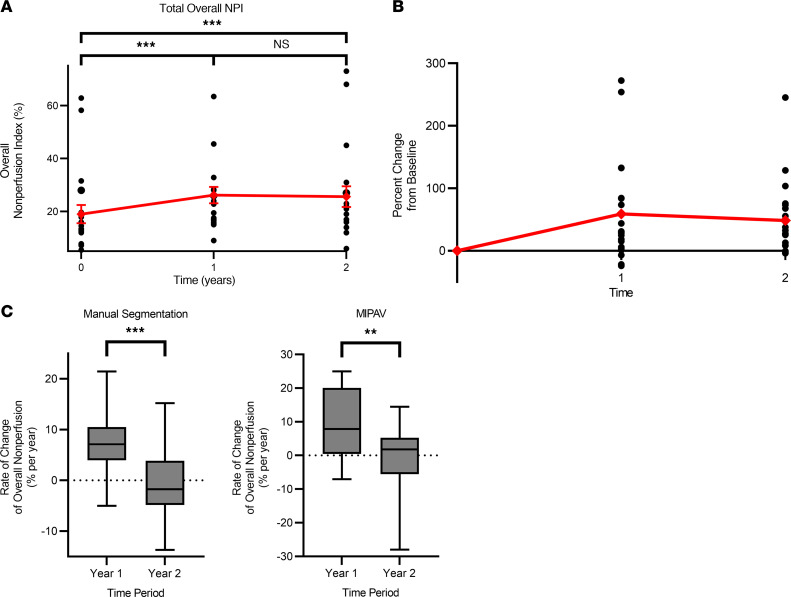
(**A**) Overall NPI for all 20 eyes, with the unadjusted mean ± SEM (red) at each time point. (**B**) Percentage of change from each patient’s respective baseline, with the mean percentage change shown by the red line at each time point. (**C**) The rate of change of nonperfusion values is represented as a box-and-whisker plot, with bounds from the 25th to 75th percentiles, median line, and whiskers ranging from minimum to maximum values. The rates of change of the nonperfusion area during years 1 and 2 were determined by the manual segmentation and guided automatic methods using MIPAV. MMRM statistical analysis: **P < 0.01 and ***P < 0.001.

Notably, 1 patient developed an allergic reaction to the fluorescein dye injection, so this patient did not undergo follow-up fluorescein angiographic imaging and was excluded from the analysis. Thus, Figure 4, A and C, and Table 3 show data on the average total nonperfusion and rate of change of total nonperfusion for the 20 eyes of the remaining 10 participants with RVCL-S. Specifically, the rate of change of the total nonperfusion area using manual segmentation was 7.22% [4.47, 9.97] per year and –0.69% [–4.06, 2.68] per year during years 1 and 2, respectively (*P* < 0.001) and, using the guided automatic method, was 9.22% [4.37, 14.1] per year and –0.69% [–5.35, 4.00] per year during years 1 and 2, respectively (*P* = 0.004). The average percentage change from baseline was 59.1% and 48.3% in year 1 and year 2, respectively (Figure 4B).

**Figure 5:**
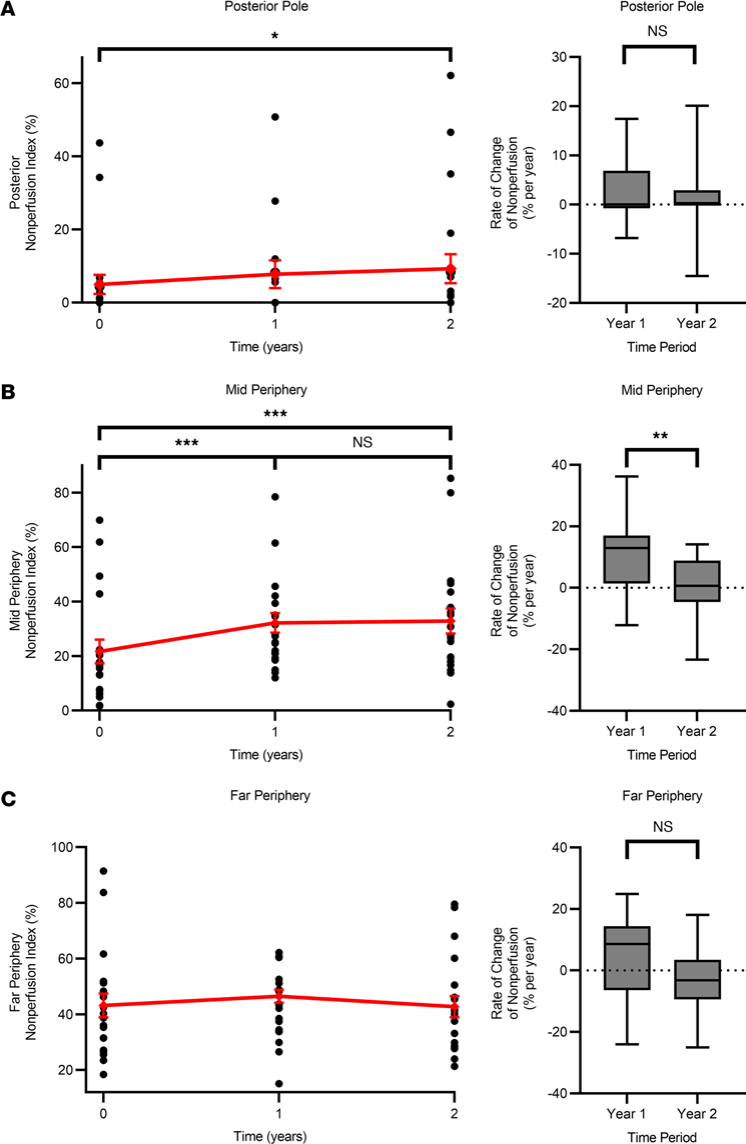
NPI values for the posterior pole, mid periphery, and far periphery. NPI (percentage) with the mean ± SEM (red) at baseline, 1 year, and 2 years and the corresponding rate of change of nonperfusion values at each retinal zone according to the manual segmentation method at (**A**) the posterior pole, (**B**) the mid periphery, and (**C**) the far periphery. Rate-of-change values are represented as a box-and-whisker plot, with bounds from the 25th to 75th percentiles, the median line, and whiskers ranging from minimum to maximum values. MMRM statistical analysis: *P < 0.05, **P < 0.01, and ***P < 0.001.

The retinal nonperfusion index (NPI) and rate of change of nonperfusion area in patients with RVCL-S were also assessed in 3 regions: posterior pole, mid periphery, and far periphery as shown graphically in Figure 5, A–C and characterized in Table 3. Specifically, the rate of change of the NPI at the mid periphery was 10.6% [5.10, 16.1] per year and 0.68% [–3.98, 5.35] per year during years 1 and 2, respectively (*P* < 0.01). The Spearman correlation coefficient of overall NPI to the posterior pole, mid periphery, and far periphery was 0.69, 0.89, and 0.77, respectively.

#### Symmetry of retinal nonperfusion between eyes.

The overall NPI for the right and left eyes of patients was averaged to determine asymmetry of progression of nonperfusion. A Wilcoxon matched-pairs signed-rank test comparing OD and OS at baseline, 1 year, and 2 years showed no difference between the right and left eyes of an individual at each time point (*P* > 0.05 [0.32, 1, 0.49, respectively]). Additionally, as found in the mixed model for repeated measures (MMRM) analysis, the change in nonperfusion over time did not greatly differ between the eyes (*P* = 0.82 for the interaction of time and eye) (Supplemental Figure 1; supplemental material available online with this article; https://doi.org/10.1172/JCI180916DS1).

#### Effect of age and sex on nonperfusion.

For the mixed-model analysis, age was added as a covariate and did not alter the significance of the effect of time on nonperfusion. To verify this, the patients were divided into 2 age groups: younger than 45 years and older than 45 years (10 eyes in each group) to determine to what degree age affects the relationship between time and nonperfusion. Through this subset analysis, the effect of time on nonperfusion was significant in both age groups for the patients on crizanlizumab (*P* < 0.05 for both age groups). Moreover, sex was added as a covariate and did not alter the significance of time on nonperfusion. Measurements of best corrected visual acuity (BCVA), CST, and nonperfusion were not significantly different between males and females (*P* > 0.05).

#### Crizanlizumab and dropouts.

Of the 7 patients who discontinued intervention, 3 patients had at least 2 fluorescein angiographic images for at least 2 of the prespecified time points. There were 4 eyes available for analysis, and a MMRM determined that the patients who dropped out of the study had 24.3% ± 9.6 % more nonperfusion than did patients on crizanlizumab (*P* = 0.0246). Specifically, the patients who dropped out had 26.7% ± 9.5 % more nonperfusion in the far periphery than did the patients who stayed on the crizanlizumab (*P* = 0.012).

## Discussion

Since RVCL-S was first identified in the 1980s, monumental strides have been made in understanding its genetic and pathophysiological underpinnings, offering innovative avenues to monitor and treat this rare, fatal disease. Advances in technologies like ultra-widefield (UWF) fundoscopy and OCT have been crucial to this increased understanding, providing insights into the effects of RVCL-S on the neurosensory retina (4). These technological breakthroughs have facilitated the ability to track disease progression of RVCL-S and the search for potential biomarkers of systemic disease progression. Notably, groundbreaking clinical trials for RVCL-S, including John Atkinson’s pioneering aclarubicin trial, have laid the foundation for clinical studies to continue the search for treatment options (10). This phase II study leverages decades of research to explore the potential of crizanlizumab as a therapeutic option for RVCL-S. Additionally, this study introduces the use of NPI as a biomarker to monitor disease progression and therapeutic response in RVCL-S retinopathy. Primarily, our findings underscore the safety of crizanlizumab in patients with RVCL-S and offer preliminary evidence of its efficacy in slowing the progression of retinal nonperfusion.

Of the 11 patients who received crizanlizumab, it is reassuring that none experienced severe adverse side effects attributable to the infusions of crizanlizumab alone. The side effects were mild and included fatigue, nausea, vomiting, and problems with stool consistency. Additionally, the side effect profile and incidence were similar to those seen in crizanlizumab studies for sickle cell pain crisis patients at the same dosages (11). Patients experiencing these symptoms were treated symptomatically, and none of the patients in the study discontinued crizanlizumab because of intolerability of the infusions, which likely contributed to the successful completion of 11 patients in the present clinical study.

In addition to safety, we performed an exploratory analysis of ophthalmic efficacy of crizanlizumab. The primary ophthalmological clinical endpoint was the extent of retinal nonperfusion and the rate of change in retinal nonperfusion of RVCL-S patients’ eyes.

Prior to this study, the quantification of nonperfusion in RVCL-S lacked a standardized methodology in the literature. Drawing from the precedent set by studies in similar nonperfusion vasculopathies like diabetic retinopathy, our approach adapted the use of the nonperfusion index (percentage) derived from fluorescein angiography (FA) for RVCL-S — what we believe to be a novel application in the context of the existing literature (12, 13). Retinal nonperfusion has been established as a key biomarker closely linked to diabetic retinopathy severity, risk of disease progression, and risk of ophthalmic complications like neovascularization (14–16). Attempts to perform similar studies in RVCL-S have been limited by the rarity of the disease. However, the use of NPI in other ophthalmic vasculopathies and in this study underscores the potential for annual monitoring of nonperfusion progression as a proxy for ophthalmic disease severity and potentially as a structural parallel to the development of cerebral lesions in RVCL-S.

**Figure 6:**
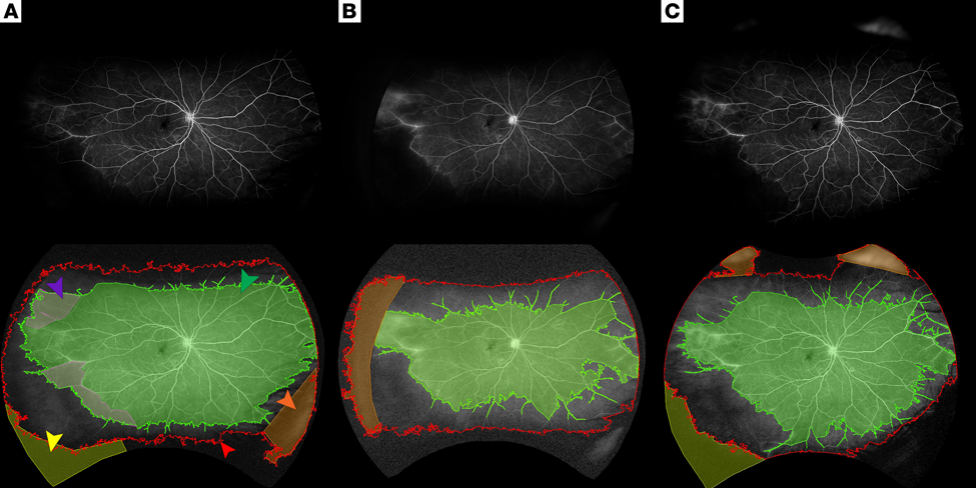
MIPAV-guided segmentation of UWF images. Original (top) and corresponding segmented (bottom) UWF images using MIPAV-guided automatic method at baseline (**A**), 1 year (**B**), and 2 years (**C**), showing the area of perfusion (green arrow), the TA outline (red arrow), the negative correction of area of perfusion (purple arrow), the negative correction of the TA (orange arrow), and the positive correction of the TA (yellow arrow).

Our manual segmentation analysis indicated a marked deviation from baseline nonperfusion at both 1 year and 2 years across the studied RVCL-S eyes. This observation aligns with the temporal pattern of new brain lesion emergence in RVCL-S, noted to occur at 6- to 12-month intervals (17). Moreover, guided automatic segmentation through medical image processing, analysis, and visualization (MIPAV) suggests that progression of nonperfusion could be appreciated qualitatively on an annual basis with the assistance of image analysis software (Figure 6, A–C).

Both manual and guided automatic analyses revealed a congruent and substantial yearly progression of retinal nonperfusion by 7.22%–9.22% during the initial year of crizanlizumab treatment and by –0.69% during the second year. The observed difference in the rates of change in nonperfusion between year 1 and year 2 under crizanlizumab treatment is noteworthy. The rate of change in nonperfusion can best be understood as the rate of decline of retinal perfusion, which was determined by using the NPI to quantify the extent of nonperfusion in RVCL-S patients’ eyes at various time points. While the rate of cognitive or visual deficits with RVCL-S can vary greatly between patients and worsen precipitously, the condition demonstrates a linear progression of brain atrophy (5). For example, previous studies that examined white matter atrophy in RVCL-S brain lesions demonstrated that white matter volume decreased linearly, and so retinal nonperfusion was anticipated to follow a similar trajectory of linear progression (17).

This linear progression followed by plateauing may suggest numerous possibilities. One possibility is that because the patients with RVCL-S continued to receive clinically appropriate interventions, treatments like anti-VEGF or PRP may have been recommended on the basis of clinical progression of RVCL-S around the second year, leading to the observed plateauing. However, no participants underwent laser photocoagulation at any point during the study, and only 2 patients received anti-VEGF treatment regularly both before and during the clinical study, suggesting that these clinical interventions may have had little effect on the observed plateauing. Another possibility may be that the assumption of linear progression may not accurately reflect the variable nature of RVCL-S vasculopathy, fluctuating between periods of stability and progression. The absence of established benchmarks for the natural progression of nonperfusion thus complicated comparative analysis. To address this gap, future research should include longitudinal studies of patients with RVCL-S not undergoing treatment.

Another promising possibility is that crizanlizumab could have reduced the rate of retinal nonperfusion, with its effect becoming structurally evident over time. By targeting P-selectin — a crucial molecule in regulating leukocyte adhesion to activated vascular endothelium — crizanlizumab might have curtailed ischemia in retinal vessels, thereby decelerating nonperfusion progression. As such, the plateauing observed in the second year of therapy might indicate a therapeutic effect. At the same time, RVCL-S participants’ vision remained unchanged at a Snellen of 20/20–20/25 at the trial’s conclusion, which is clinically important to patients who greatly value the preservation of their central vision. Given crizanlizumab’s role in preventing vaso-occlusive episodes for patients with sickle cell disease, the observed post-treatment changes may indicate the potential of crizanlizumab for alleviating RVCL-S retinal vasculopathy. Further studies are needed to discern whether this potential therapeutic effect is sustainable and if it becomes more pronounced with extended treatment, potentially even restoring perfusion.

Additionally, we used retinal zone segmentation was used for a nuanced analysis of how different retinal zones are affected. The prevailing observation that nonperfusion starts peripherally and advances centrally is corroborated by our findings of a decreasing NPI from the far periphery to the posterior pole (4). This detailed segmentation revealed clinically relevant insights, particularly that mid-periphery NPI changes (*r* = 0.89) more closely mirrored overall NPI shifts compared with the posterior pole and far periphery (*r* = 0.69 and *r* = 0.77, respectively). Moreover, the mid periphery demonstrated the same plateauing in the rate of change during the second year, unlike the posterior pole or far periphery (Figure 5B). It was initially hypothesized that the far periphery would be most correlated to total changes in the NPI because ischemia progresses from the periphery to the center. However, because the far periphery is both the first to be affected and is the region most confounded by artifact inherent to UWF imaging, it is reasonable that the mid periphery would be more correlated to total changes in nonperfusion. Thus, focusing on the mid periphery of the retina for monitoring RVCL-S nonperfusion progression might offer a more accurate reflection of overall nonperfusion changes and response to therapy compared with the other retinal zones.

Secondary endpoints of visual function and structure revealed that visual acuity and IOP remained stable over the 2 years of treatment. While neovascularization of the iris is a possible complication due to the vasculopathy of RVCL-S, this suggests that, in the absence of pressure-affecting pathologies such as neovascular glaucoma, IOP is not significantly affected by RVCL-S itself over time. CST, also analyzed through logarithmic transformation to address skewed distributions, showed no significant change across the study for this cohort, which corroborates previous observations that central retinal atrophy is not a major manifestation early in the disease process (4). Additionally, further comparison with the normative database similarly demonstrated that CST values at baseline and at the study’s conclusion were not different from those of healthy controls, suggesting that CST may not be markedly affected in the pathophysiology of RVCL-S.

RVCL-S is typically thought of as a systemic disease that is presumed to cause symmetric vasculopathy rather than affecting 1 eye preferentially. Our findings showed no significant difference in retinal nonperfusion between eyes at baseline, year 1, and year 2. Additionally, the progression of nonperfusion over time was similar between eyes, suggesting that retinal nonperfusion did not asymmetrically manifest in patients with RVCL-S taking crizanlizumab. However, it is important to note that disease progression can vary considerably among patients, with some experiencing more severe ophthalmic complications in 1 eye than in the other. As such, a personalized, eye-specific management approach is recommended, as patients can have worse BCVA in 1 eye or unilateral macular edema, as seen in some of the participants in this study. Age, as a covariate in the mixed-model analysis, did not significantly alter the time effect on nonperfusion, nor did analyses segregated by age groups (<45 years and >45 years of age), suggesting uniform progression across ages. This finding suggests that the observed severity of RVCL-S at older ages may have resulted more from cumulative vascular damage than from an accelerated rate of progression at older ages.

In a post hoc analysis, patients who withdrew from the study and had at least 2 fluorescein angiographic images showed 24.3% higher overall nonperfusion and a 26.7% increase in nonperfusion in the far periphery compared with those who completed the study. This suggests that the patients who dropped out of the study had more severe disease manifestations, which may have influenced their decision to withdraw, although this is speculative, given the small sample size. However, a comparison of ongoing participants with participants who withdrew — considering the unequal sample sizes and nonrandom withdrawal reasons — introduced potential confounders and limited appropriate statistical analysis. Though the comparison was limited for these reasons, it is intriguing that the average time from *TREX1* mutation diagnosis, which presumably mirrored disease burden, was 5 years in those who withdrew compared with 1.6 years for those who completed the study. As such, disease severity likely played a major role in the patients’ ability to adhere to crizanlizumab infusions.

In this study, there are notable limitations that warrant cautious interpretation. The small sample size and lack of a control group, given the study’s exploratory nature focusing on safety and preliminary efficacy, pose noteworthy constraints to determining efficacy. However, this is a rare disease with fewer than 25–40 families diagnosed worldwide, creating notable constraints on efforts to increase the sample size in future studies. Additionally, there is a risk of bias with manual segmentation, which is prone to variability because of the subjective interpretation of the grader. Still, the guided automatic method that was used showed comparable rates of progression when compared with the manual segmentation method, bolstering confidence in the analyses gleaned from the manual quantification of nonperfusion.

Despite these limitations, this clinical study is important, as it represents, to our knowledge, the largest clinical observational study to date exploring potential therapeutic options for patients with RVCL-S who currently have no disease-modifying treatment options. The implications of a well-tolerated treatment option for this rare and fatal disease are certainly clinically relevant for these patients. Moreover, the potential efficacy of crizanlizumab in the treatment of RVCL-S provides insight into the possible role of leukocyte adhesion and recruitment in the pathology of similar small-vessel diseases. The implication that inhibition of key steps of cell adhesion may mitigate ischemic burden could extend to other similar small-vessel retinal diseases, inspiring future treatments that target different cell adhesion pathways in a broader range of small-vessel diseases. In the future, we hope to evaluate the potential role of the retina as a biomarker of the neurological aspects of RVCL-S by correlating our rates of retinal nonperfusion with rates of white matter atrophy and brain lesion progression and exploring connections between retinal nonperfusion and neurological deterioration. Additionally, patients with RVCL-S commonly present with visual complaints early in the disease process, prior to cognitive impairment. Future investigations are needed to more fully evaluate the role of the retina as an early biomarker to facilitate early diagnosis and treatment, before substantial blindness and cognitive decline occur, as has been investigated in other neurodegenerative diseases (18, 19).

In summary, this study presents promising results of crizanlizumab as a potential therapy to slow the progression of this rare and fatal disease, and our findings warrant larger and more extensive clinical trials of crizanlizumab for RVCL-S with a more robust clinical design.

## Methods

### Sex as a biological variable

Our study examined male and female participants, as both men and women were eligible for this trial, and findings were similar for both sexes.

### Study participants

Enrollment of participants occurred between January 2021 and December 2021. Inclusion criteria included a confirmed diagnosis of RVCL-S by the *TREX1* gene test that utilizes next-generation sequencing technology to detect genomic variants in the coding regions and adjacent intronic bases within the *TREX1* gene. Participants needed to be 25 years of age or older with imaging evidence of RVCL-S in the brain or eye at the time of study registration, have normal hematologic function with a WBC count of greater than 4 × 10^9^/L, an absolute neutrophil count of greater than 1.5 × 10^9^/L, and a platelet count of greater than 100 × 10^9^/L, and, if female, agree to refrain from becoming pregnant while on the study drug and 3 months after discontinuation by using adequate contraceptive measures. Key exclusion criteria included acute bacterial, fungal, or viral infection, known HIV, untreated latent tuberculosis (TB), active hepatitis B or C infection, zoster infection, pregnancy and/or breastfeeding, known hypersensitivity to the study agents, recent investigational drug or monoclonal antibody use, liver function tests (LFTs) higher than 3 times the upper limit of normal within the last 30 days, and anticoagulation agent use within the last 30 days.

### Procedures

Enrolled participants received crizanlizumab via intravenous infusion at a dose of 5 mg/kg at specified intervals over 24 months, with initial doses in weeks 1 and 3, followed by monthly administration after week 7. Locations for infusion varied according to patient preference, with mandatory follow-up visits at Washington University at months 1, 6, 12, 18, and 24. Crizanlizumab infusions were continued unless considered detrimental to the patient’s health or the patient decided to withdraw from the study. Patients were monitored for 30 minutes after infusion for any adverse reactions, with specific protocols for COVID-19 screening and management of adverse events, including drug-induced liver injury, as per the study protocol.

### Standard ophthalmic examination

Standard ophthalmic examination included BCVA, IOP, confrontation visual fields, extraocular eye movements, slit lamp examination, fundus biomicroscopy, and indirect ophthalmoscopy of the fundus.

### Fundus photography and FA

The Optos widefield scanning laser ophthalmoscope (SLO) was used for fundus imaging, as established in previous methodologies. This imaging methodology produced color fundus, autofluorescence, and fluorescein angiographic images for subsequent analysis. For these procedures, eye dilation was achieved using 1% tropicamide and 2.5% phenylephrine. In the FA protocol, 10% sodium fluorescein was then administered intravenously, and a sequence of images was captured at specific time points: baseline, early arteriovenous phase (0–60 seconds after injection), and late arteriovenous phase (3–5 minutes after injection) (4).

Retina specialists undertook a comprehensive evaluation of all fluorescein angiograms and subsequent analysis. The graders reached a consensus on the final interpretation.

### FA evaluation

#### Definition of retinal zones.

The methodology for measuring retinal nonperfusion in this study was adapted from Silva et al. (12). In summary, all UWF images underwent stereographic projection, and the retinal zones (posterior pole, mid periphery, and far periphery) were defined on the basis of locations of the fovea and optic nerve head. Each UWF image was registered to ensure precise pixel-level segmentation and demarcation of the individual retinal zones. Utilizing the annotation tool within the Optos software, a free-hand line was drawn from the foveal center to demarcate distances of 10 mm and 15 mm. Subsequently, circles with the specified radii were created to digitally overlay 2 concentric circles with radii of 10 mm and 15 mm onto each image, thereby dividing each fluorescein angiographic image into 3 designated retinal zones. The areas enclosed by the concentric circles were then recorded and used to calculate the total gradable area (TA) for the posterior, mid periphery, and far periphery zones in each image.

#### Quantification of retinal nonperfusion area at retinal zones and the NPI.

Nonperfusion areas and TA for each eye and each retinal zone were calculated in square millimeters by summing the size of all pixels within the appropriate designated area using a proprietary tool (Optos) that implements DICOM Supplement 173.11. Briefly, the size of an individual pixel was individually defined by its location and was calculated using spherical trigonometry after projecting it back onto a spherical surface, allowing an accurate estimation of the retinal area (mm^2^) independent of peripheral image distortion (12, 20).

**Figure 7:**
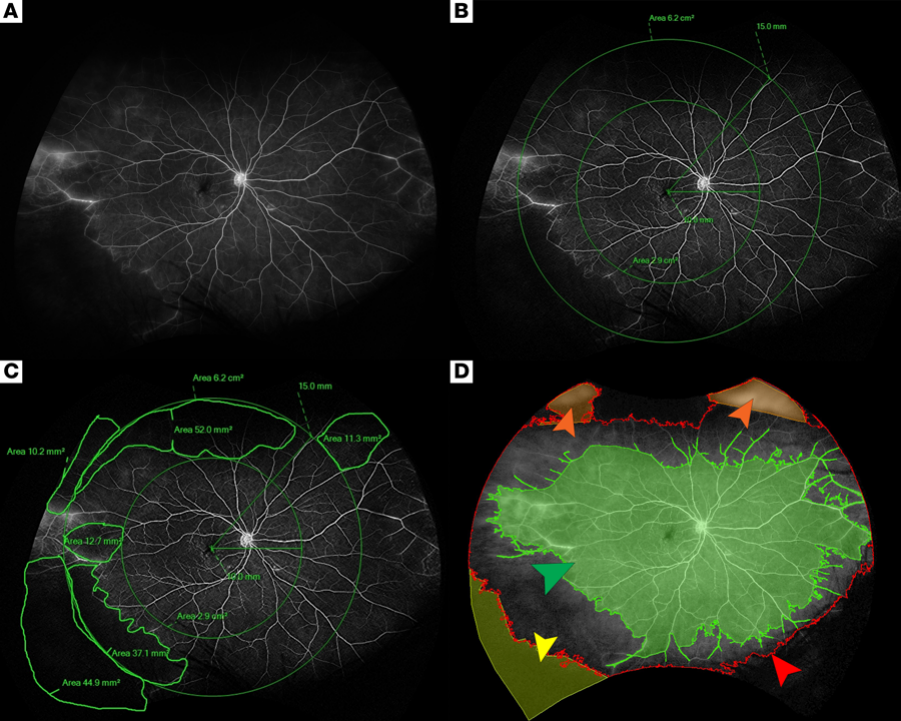
Example image segmentation for a patient with RVCL-S. (**A**) UWF fundoscopic image without segmentation. (**B**) UWF with concentric circles of radii 10 mm and 15 mm from the foveal avascular zone and areas enclosed delineate the retinal zones of the posterior pole (*r* between 0 mm and 10 mm), mid periphery (*r* between 10 mm and 15 mm), and far periphery (*r* >15 mm). (**C**) Manual segmentation of nonperfusion utilizing Optos software that quantifies the desired masked area. (**D**) MIPAV-guided automated method (also shown in Figure 6C) of quantification measuring the area of perfusion (green arrow), the outline of the TA (red arrow), negative correction of the TA (orange arrows), and positive correction of the TA (yellow arrow).

The extent and distribution of nonperfusion were assessed by dividing each image into 3 retinal zones. As described previously, each image was registered to create concentric zones centered on the fovea to delineate the extent of the posterior pole (<10 mm), mid periphery (10–15 mm), and far periphery (>15 mm) (Figure 7, A and B). Based on measurements made on a Navarro model eye, posterior pole, mid periphery, and far periphery comprised 32%, 35%, and 33%, respectively, of the total retinal surface area of each fluorescein angiographic image. This distribution is expected to vary due to imaging artifacts, mainly affecting the superior and inferior far periphery (12).

For each eye, the extent of nonperfusion at each retinal zone (posterior, mid periphery, and far periphery) was determined by summing up the pixels determined to be nonperfusion in that specific retinal zone and dividing by the corresponding TA to determine the NPI, expressed as a percentage, in each retinal zone (Figure 7C). Subsequently, the nonperfusion areas from all retinal zones were aggregated to calculate the total nonperfusion area, which was then divided by the TA to yield the overall NPI (given as a percentage). This process was repeated for 3 images of the same eye at each time point, and the results were averaged to provide an estimate of the extent of nonperfusion in each zone.

Segmentation and grading were performed by 1 grader under the guidance of a retina specialist, which included extensive training on identifying areas of nonperfusion. The definition of retinal nonperfusion was adapted from the Early Treatment Diabetic Retinopathy Study (ETDRS) FA grading protocol and the Standard Care versus Corticosteroid for Retinal Vein Occlusion (SCORE) study (21). Shadows were differentiated from nonperfused areas by the presence of retinal vessels and the lack of a clear boundary between perfused and nonperfused retinal regions. Areas affected by artifacts such as eyelashes were excluded from analysis (22). The image segmentations were reviewed in detail for accuracy, verification, and standardization by a retina specialist with expertise in analyzing UWF FA images. Images were not masked because of the necessity to use patient information to locate and grade images using proprietary software from Optos. Intragrader reliability was determined by Pearson’s correlation of all patients’ first and third measurements for nonperfusion of the total retina, posterior pole, mid periphery, and far periphery and were 0.93, 0.97, 0.86, and 0.68, respectively.

In addition to manual segmentation, images were segmented through guided automatic segmentation using MIPAV software (https://mipav.cit.nih.gov/) levelset VOI tool to determine the TA and total area of perfusion in voxels, which could then be used to calculate the nonperfusion area and the overall NPI for each image. Each automatic segmentation was manually corrected, if necessary, through negative correction — subtracting an area that was incorrectly included, or through positive correction — adding an area that was incorrectly excluded (Figure 7D). The best image for each eye was utilized for guided automatic segmentation. Given the nature of the software, the guided automatic segmentation tool was only able to calculate the NPI overall and not according to the prespecified retinal zones. Moreover, nonperfusion area calculations from the MIPAV-guided automatic method utilized voxels rather than DICOM Supplement 173.11, so MIPAV measurements were subject to peripheral image distortion. As such, while MIPAV point estimates of overall NPI may not accurately reflect the proportion of nonperfusion at each time point, this method can be used to quantify changes in overall nonperfusion and do so with less potential bias than the manual segmentation method.

#### OCT.

OCT imaging of the optic disc, macula, and vasculature was conducted by a certified operator using the Heidelberg Retina Angiograph HRA plus OCT Spectralis system (Heidelberg Engineering) in the retina clinic of the Center for Outpatient Health at Washington University in St. Louis. OCT scans were performed at baseline and at the 2-year follow-up, following a protocol previously performed for image acquisition (4). In brief, measurements were automated with the manufacturer’s software, utilizing an infrared beam of superluminescent diode to capture high-quality OCT images. Automated retinal segmentation was used to generate thickness values for different retinal layers in accordance with the ETDRS macular map, and scans were carefully examined to ensure accurate segmentation. The collected data included CST measurements obtained from the Spectralis OCT at both baseline and the 2-year time point. CST values (μm) were transformed logarithmically to generate a more normalized distribution for subsequent data analyses (23). Participant’s right eye CST values at baseline and year 2 were compared with a normative database of 297 patients using the Mann-Whitney *U* test (24).

### Statistics

Data analyses were conducted using R Studio (version R-4.2.1) and GraphPad Prism 9.50 (GraphPad Software). Descriptive statistics were used to summarize the data as appropriate. Both parametric and nonparametric tests were utilized as appropriate per analysis requirements. As per Silva et al., to account for the modeling of zero values, all overall and posterior NPIs were transformed by T.NPI = [NPI × (*n* − 1) + 0.5]/*n*], where *n* = 20 (total number of eyes) and transformed back where appropriate for interpretation (12). MMRM analysis was performed for longitudinally correlated eye data, introducing random effects to account for within-patient longitudinal correlation and inter-eye correlation (25). Time was incorporated as a fixed variable, with age and sex added as fixed effects in select analyses. A *P* value of less than 0.05 was considered significant. Unless otherwise stated, the mixed model used for analysis included only time as a fixed variable.

### Study approval

This study was approved by IRB of Washington University in St. Louis (approval no. 202008079) and registered with ClinicalTrials.gov as NCT04611880 on November 2, 2020. The study was conducted according to the tenets of the Declaration of Helsinki. Health Insurance Portability and Accountability Act provisions were adhered to, the risks and benefits were discussed with each individual participant, and written informed consent was obtained prior to beginning any infusion. Participants received monthly infusions at either Washington University or the University of Pennsylvania. Except for adverse event recording and documentation of infusions, all data collection regarding eye disease monitoring occurred at Washington University. Men, women, and members of all races and ethnic groups were eligible for this trial.

### Data availability

The source data for data points shown in graphs and values behind means are provided in the Supporting Data Values file. Additional information can be obtained upon request to the corresponding author.

## Author contributions

RSA, ALF, and PKR were responsible for research conception, research design, investigation, and methodology. ALF, RSA, and PKR were responsible for acquiring data and providing clinical care. WXW was responsible for data acquisition and organization, analysis methodology, and implementation. WXW, DS, and RSA were involved in the writing and revision of the final manuscript. All authors read, edited, and approved the manuscript.

## Supplementary Material

Supplemental data

ICMJE disclosure forms

Supporting data values

## Figures and Tables

**Table 2:**
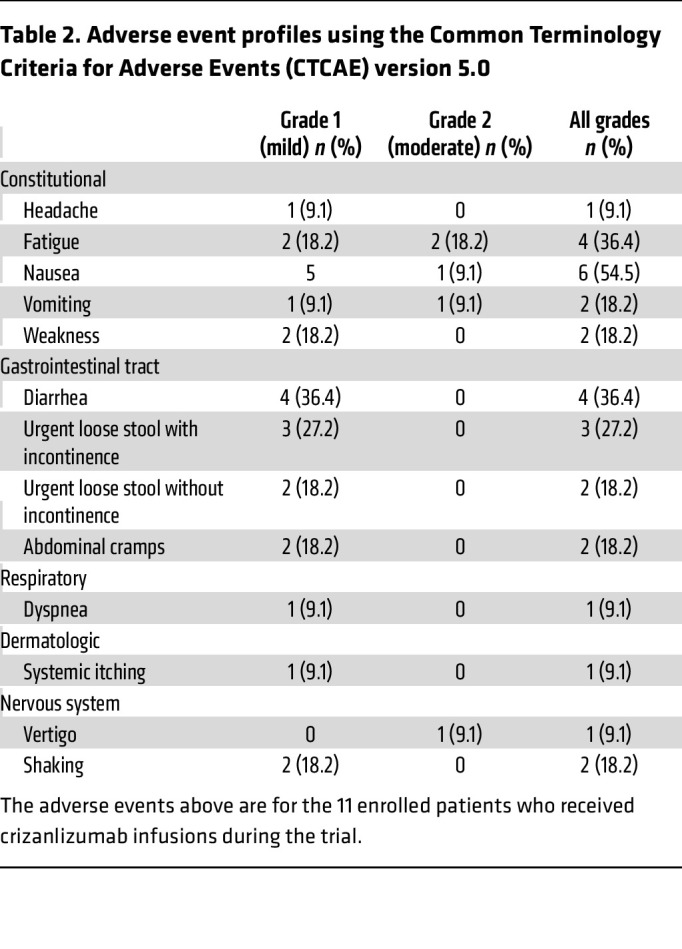
Adverse event profiles using the Common Terminology Criteria for Adverse Events (CTCAE) version 5.0

**Table 1:**
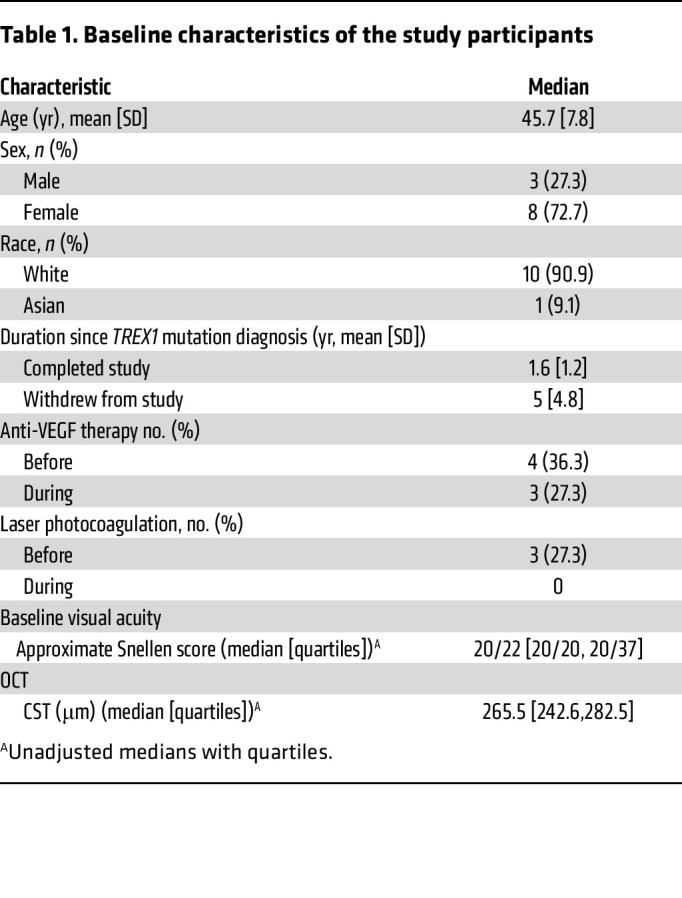
Baseline characteristics of the study participants

**Table 3:**
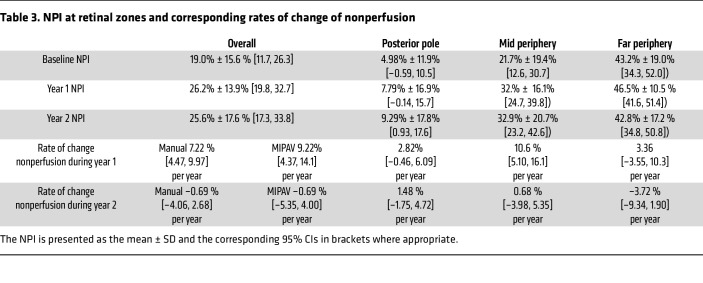
NPI at retinal zones and corresponding rates of change of nonperfusion

## Version 1 06/17/2024

Electronic publication
