# Crizanlizumab for retinal vasculopathy with cerebral leukoencephalopathy in a phase II clinical study

**DOI:** 10.1172/JCI189023

**Published:** 2024-12-16

**Authors:** Wilson X. Wang, Dan Spiegelman, P. Kumar Rao, Rennie L. Rhee, Andria L. Ford, Jonathan J. Miner, Rajendra S. Apte

**Affiliations:** 1 John F. Hardesty, MD Department of Ophthalmology and Visual Sciences, Washington University School of Medicine, St. Louis, Missouri, USA.; 2 Department of Medicine and RVCL Research Center, University of Pennsylvania Perelman School of Medicine, Philadelphia, Pennsylvania, USA.; 3 Department of Neurology, Washington University School of Medicine, St. Louis, Missouri, USA.; 4 Departments of Medicine and Microbiology, Colton Center for Autoimmunity, and RVCL Research Center, University of Pennsylvania Perelman School of Medicine, Philadelphia, Pennsylvania, USA.; 5 Department of Medicine, Washington University, St. Louis, Missouri, USA.

Original citation: *J Clin Invest*. 2024;134(12):e180916. https://doi.org/10.1172/JCI180916

Citation for this corrigendum: *J Clin Invest*. 2024;134(24):e189023. https://doi.org/10.1172/JCI189023

Jonathan J. Miner and Rennie Rhee were omitted from the author list. Dr. Miner designed, initiated, obtained funding for, and oversaw the clinical trial while serving at Washington University, and continued to work on it after relocating his laboratory and practice to the University of Pennsylvania Perelman School of Medicine (Penn), the clinical trial’s second site. Dr. Miner is cocorresponding author for this article. Dr. Miner declares compensation by Novartis for an educational P-selectin lecture presented to Novartis staff prior to initiation of the clinical trial. Dr. Rennie Rhee was the principal investigator at Penn. The HTML and PDF versions of this article have been updated with the correct author and affiliation lists.

In addition, information regarding the primary endpoint was omitted from the discussion. Progression in brain lesions on MRI was designated as the primary endpoint of the “Crizanlizumab for RVCL” clinical trial, and the methodology for assessing retinal nonperfusion was developed as a post hoc analysis after the clinical trial was completed. In RVCL-S patients receiving crizanlizumab, we have observed clear progression of brain lesions based on MRI results. A detailed analysis of the brain MRI results, including information about the rate of disease progression, is in preparation.

The authors regret the errors.

## Version 1 12/16/2024

Electronic publication

