# Supplementary material for: Crizanlizumab for retinal vasculopathy with cerebral leukoencephalopathy in a phase II clinical study
Source: J Clin Invest. 2024 May 7;134(12):e180916. doi: 10.1172/JCI180916 (PMC11178534; doi:10.1172/JCI180916)
Supplement: Supplemental data [file jci-134-180916-s106.pdf]

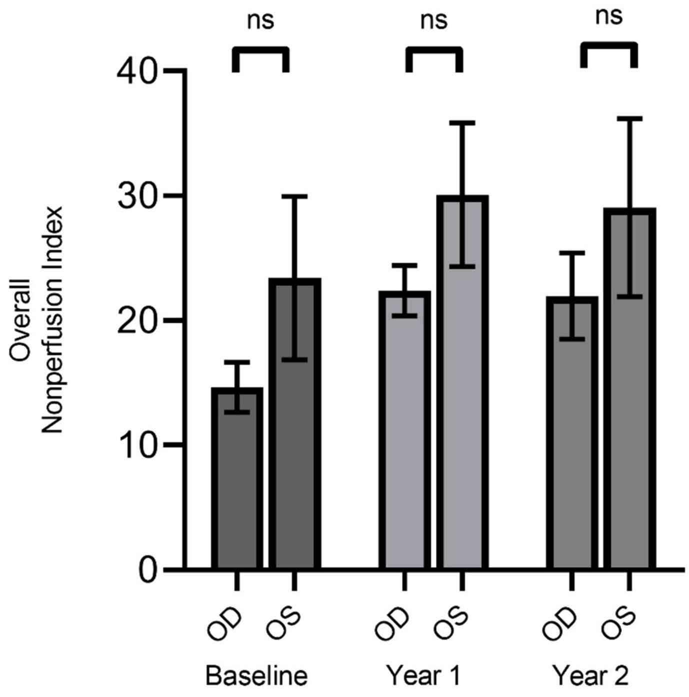

Supplemental Figure 1. Comparison of mean NPI between the right and left eyes of RVCL-S individuals. ns: not significant.
