# Supplementary material for: Crizanlizumab for retinal vasculopathy with cerebral leukoencephalopathy in a phase II clinical study
Source: J Clin Invest. 2024 May 7;134(12):e180916. doi: 10.1172/JCI180916 (PMC11178534; doi:10.1172/JCI180916)
Supplement: ICMJE disclosure forms [file jci-134-180916-s107.pdf]

ICMJE FORM

**Date:**	3/5/2024
**Your Name:**	Wilson X. Wang
**Manuscript Title:**	Crizanlizumab for Retinal Vasculopathy with Cerebral Leukoencephalopathy in a Phase 2 clinical study
**US-based Author (if yes, you must fill out Open Payment section below):**	NO
**Manuscript Number (if known):**	

Ophthalmology complies with the position of the International Committee of Medical Journal Editors (ICMJE) on "Conflict of Interest." Conflict of interest for authors is defined as "financial and other conflicts of interest that might bias their work."

In the interest of transparency, we ask you to disclose all relationships/activities/interests listed below that are related to the content of your manuscript. "Related" means any relation with for-profit or not-for-profit third parties whose interests may be affected by the content of the manuscript. Disclosure represents a commitment to transparency and does not necessarily indicate a bias. If you are in doubt about whether to list a relationship/activity/interest, it is preferable that you do so.

The author's relationships/activities/interests should be defined broadly. For example, if your manuscript pertains to the epidemiology of hypertension, you should declare all relationships with manufacturers of antihypertensive medication, even if that medication is not mentioned in the manuscript.

In item #1 below, report all support for the work reported in this manuscript without time limit. For all other items, the time frame for disclosure is the past 36 months.

		**Name all entities with whom you have this relationship or indicate none (add rows as needed)**	**Specifications/Comments (e.g., if payments were made to you or to your institution)**
		Time frame: Since the initial planning of the work	
1	All support for the present manuscript (e.g., funding, provision of study materials, medical writing, article processing charges, etc.) No time limit for this item.	☐ None	
		Washington University MD5 Program: DeNardo Education & Research Foundation Grant	
			Click the tab key to add additional rows.
		Time frame: past 36 months	
**2**	Grants or contracts from any entity (if not indicated in item #1 above).	☒ **None**	

		Name all entities with whom you have this relationship or indicate none (add rows as needed)	Specifications/Comments (e.g., if payments were made to you or to your institution)
**3**	Royalties or licenses	☒ **None**	
**4**	Consulting fees	☒ **None**	
**5**	Payment or honoraria for lectures, presentations, speakers bureaus, manuscript writing or educational events	☒ **None**	
**6**	Payment for expert testimony	☒ **None**	
**7**	Support for attending meetings and/or travel	☒ **None**	
**8**	Patents planned, issued or pending	☒ **None**	
**9**	Participation on a Data Safety Monitoring Board or Advisory Board	☒ **None**	
**10**	Leadership or fiduciary role in other board,	☒ **None**	

		Name all entities with whom you have this relationship or indicate none (add rows as needed)	Specifications/Comments (e.g., if payments were made to you or to your institution)
	society, committee or advocacy group, paid or unpaid		
**11**	Stock or stock options	☒ **None**	
**12**	Receipt of equipment, materials, drugs, medical writing, gifts or other services	☒ **None**	
**13**	Other financial or non-financial interests	☒ **None**	

Please place an "X" next to the following statement to indicate your agreement:

☒ I certify that I have answered every question and have not altered the wording of any of the questions on this form.

FOR US-BASED AUTHORS ONLY>>> OPEN PAYMENTS

For US-based authors (i.e., those with a US National Provider Identifier [NPI]), it is now required they look up their Open Payments record (past 2 years only) to confirm that it matches what is reported on the ICMJE Form. If there are discrepancies, the co-author must provide a brief explanation on the ICMJE Form along with the Open Payment URL person record (e.g., https://openpaymentsdata.cms.gov/physician/1316209). To learn more about this new requirement, please refer to Van Gelder RN and Siegfried CJ. ROI, COI, and the Ethical Obligations of Journals. Ophthalmology. 2022; 129: 602-604.

Open Payments URL: Click or tap here to enter text.

Match Disclosure Form? YES/NO

If no, please briefly explain discrepancy: Click or tap here to enter text.

ICMJE DISCLOSURE FORM

**Date:**	3/2/2024
**Your Name:**	Dan Spiegelman
**Manuscript Title:**	Crizanlizumab for Retinal Vasculopathy with Cerebral Leukoencephalopathy in a Phase 2 clinical study
**Manuscript Number (if known):**	Click or tap here to enter text.

In the interest of transparency, we ask you to disclose all relationships/activities/interests listed below that are related to the content of your manuscript. "Related" means any relation with for-profit or not-for-profit third parties whose interests may be affected by the content of the manuscript. Disclosure represents a commitment to transparency and does not necessarily indicate a bias. If you are in doubt about whether to list a relationship/activity/interest, it is preferable that you do so.

The author's relationships/activities/interests should be defined broadly. For example, if your manuscript pertains to the epidemiology of hypertension, you should declare all relationships with manufacturers of antihypertensive medication, even if that medication is not mentioned in the manuscript.

In item #1 below, report all support for the work reported in this manuscript without time limit. For all other items, the time frame for disclosure is the past 36 months.

		**Name all entities with whom you have this relationship or indicate none (add rows as needed)**	**Specifications/Comments (e.g., if payments were made to you or to your institution)**
		Time frame: Since the initial planning of the work	
1	All support for the present manuscript (e.g., funding, provision of study materials, medical writing, article processing charges, etc.) No time limit for this item.	☒ None	
			Click the tab key to add additional rows.
		Time frame: past 36 months	
**2**	Grants or contracts from any entity (if not indicated in item #1 above).	☒ **None**	
**3**	Royalties or licenses	☒ **None**	

		Name all entities with whom you have this relationship or indicate none (add rows as needed)	Specifications/Comments (e.g., if payments were made to you or to your institution)
**4**	Consulting fees	☒ **None**	
**5**	Payment or honoraria for lectures, presentations, speakers bureaus, manuscript writing or educational events	☒ **None**	
**6**	Payment for expert testimony	☒ **None**	
**7**	Support for attending meetings and/or travel	☒ **None**	
**8**	Patents planned, issued or pending	☒ **None**	
**9**	Participation on a Data Safety Monitoring Board or Advisory Board	☒ **None**	
**10**	Leadership or fiduciary role in other board, society, committee or advocacy group, paid or unpaid	☒ **None**	

		Name all entities with whom you have this relationship or indicate none (add rows as needed)	Specifications/Comments (e.g., if payments were made to you or to your institution)
**11**	Stock or stock options	☒ **None**	
**12**	Receipt of equipment, materials, drugs, medical writing, gifts or other services	☒ **None**	
**13**	Other financial or non-financial interests	☒ **None**	

Please place an "X" next to the following statement to indicate your agreement:

☒ I certify that I have answered every question and have not altered the wording of any of the questions on this form.

ICMJE FORM

**Date:**	3/3/2024
**Your Name:**	Prabakar Kumar Rao
**Manuscript Title:**	Crizanlizumab for Retinal Vasculopathy with Cerebral Leukoencephalopathy in a Phase 2 clinical study
**US-based Author (if yes, you must fill out Open Payment section below):**	YES
**Manuscript Number (if known):**	

Ophthalmology complies with the position of the International Committee of Medical Journal Editors (ICMJE) on "Conflict of Interest." Conflict of interest for authors is defined as "financial and other conflicts of interest that might bias their work."

In the interest of transparency, we ask you to disclose all relationships/activities/interests listed below that are related to the content of your manuscript. "Related" means any relation with for-profit or not-for-profit third parties whose interests may be affected by the content of the manuscript. Disclosure represents a commitment to transparency and does not necessarily indicate a bias. If you are in doubt about whether to list a relationship/activity/interest, it is preferable that you do so.

The author's relationships/activities/interests should be defined broadly. For example, if your manuscript pertains to the epidemiology of hypertension, you should declare all relationships with manufacturers of antihypertensive medication, even if that medication is not mentioned in the manuscript.

In item #1 below, report all support for the work reported in this manuscript without time limit. For all other items, the time frame for disclosure is the past 36 months.

		**Name all entities with whom you have this relationship or indicate none (add rows as needed)**	**Specifications/Comments (e.g., if payments were made to you or to your institution)**
		Time frame: Since the initial planning of the work	
1	All support for the present manuscript (e.g., funding, provision of study materials, medical writing, article processing charges, etc.) No time limit for this item.	☐ None	
		Research to Prevent Blindness	Institution
			Click the tab key to add additional rows.
		Time frame: past 36 months	
**2**	Grants or contracts from any entity (if not indicated in item #1 above).	☒ **None**	

		Name all entities with whom you have this relationship or indicate none (add rows as needed)	Specifications/Comments (e.g., if payments were made to you or to your institution)
**3**	Royalties or licenses	☒ **None**	
**4**	Consulting fees	☒ **None**	
**5**	Payment or honoraria for lectures, presentations, speakers bureaus, manuscript writing or educational events	☐ **None**	
		VRS Foundation Annual Update	Honoraria payment made directly to me
**6**	Payment for expert testimony	☒ **None**	
**7**	Support for attending meetings and/or travel	☐ **None**	
		VRS Annual Update	Payment for hotel stay made directly to hotel on my behalf
**8**	Patents planned, issued or pending	☒ **None**	
**9**	Participation on a Data Safety Monitoring Board or Advisory Board	☒ **None**	
**10**	Leadership or fiduciary role in other board,	☒ **None**	

		Name all entities with whom you have this relationship or indicate none (add rows as needed)	Specifications/Comments (e.g., if payments were made to you or to your institution)
	society, committee or advocacy group, paid or unpaid		
**11**	Stock or stock options	☒ **None**	
**12**	Receipt of equipment, materials, drugs, medical writing, gifts or other services	☒ **None**	
**13**	Other financial or non-financial interests	☐ **None**	
		CEO Hark Vision – make low cost slit lamp cameras	Payment directly to me

Please place an "X" next to the following statement to indicate your agreement:

☒ I certify that I have answered every question and have not altered the wording of any of the questions on this form.

FOR US-BASED AUTHORS ONLY>>> OPEN PAYMENTS

For US-based authors (i.e., those with a US National Provider Identifier [NPI]), it is now required they look up their Open Payments record (past 2 years only) to confirm that it matches what is reported on the ICMJE Form. If there are discrepancies, the co-author must provide a brief explanation on the ICMJE Form along with the Open Payment URL person record (e.g., https://openpaymentsdata.cms.gov/physician/1316209). To learn more about this new requirement, please refer to Van Gelder RN and Siegfried CJ. ROI, COI, and the Ethical Obligations of Journals. Ophthalmology. 2022; 129: 602-604.

**Open Payments URL:**	https://openpaymentsdata.cms.gov/physician/688771
**Match Disclosure Form?**	YES
**If no, please briefly explain discrepancy:**	Click or tap here to enter text.

ICMJE FORM

**Date:**	3/2/2024
**Your Name:**	Andria Ford
**Manuscript Title:**	Crizanlizumab for Retinal Vasculopathy with Cerebral Leukoencephalopathy in a Phase 2 clinical study
**US-based Author (if yes, you must fill out Open Payment section below):**	YES
**Manuscript Number (if known):**	

Ophthalmology complies with the position of the International Committee of Medical Journal Editors (ICMJE) on "Conflict of Interest." Conflict of interest for authors is defined as "financial and other conflicts of interest that might bias their work."

In the interest of transparency, we ask you to disclose all relationships/activities/interests listed below that are related to the content of your manuscript. "Related" means any relation with for-profit or not-for-profit third parties whose interests may be affected by the content of the manuscript. Disclosure represents a commitment to transparency and does not necessarily indicate a bias. If you are in doubt about whether to list a relationship/activity/interest, it is preferable that you do so.

The author's relationships/activities/interests should be defined broadly. For example, if your manuscript pertains to the epidemiology of hypertension, you should declare all relationships with manufacturers of antihypertensive medication, even if that medication is not mentioned in the manuscript.

In item #1 below, report all support for the work reported in this manuscript without time limit. For all other items, the time frame for disclosure is the past 36 months.

		**Name all entities with whom you have this relationship or indicate none (add rows as needed)**	**Specifications/Comments (e.g., if payments were made to you or to your institution)**
		Time frame: Since the initial planning of the work	
1	All support for the present manuscript (e.g., funding, provision of study materials, medical writing, article processing charges, etc.) No time limit for this item.	☐ None	
		Clayco foundation	Payments made to the institution, Washington University
		Novartis	Supplied the drug being studied, crizanlizumab, free of charge
			Click the tab key to add additional rows.
		Time frame: past 36 months	
**2**	Grants or contracts from any entity (if not indicated in item #1 above).	☒ **None**	
		NIH NINDS RF1	Payments made to the institution, Washington University
		NIH NHLBI R01	Payments made to the institution, Washington University
		NIH NINDS U01	Payments made to the institution, Washington University

		Name all entities with whom you have this relationship or indicate none (add rows as needed)	Specifications/Comments (e.g., if payments were made to you or to your institution)
**3**	Royalties or licenses	☒ **None**	
**4**	Consulting fees	☒ **None**	
**5**	Payment or honoraria for lectures, presentations, speakers bureaus, manuscript writing or educational events	☐ **None**	
		Pfizer	2022, 2023 – Advisory Board; Payments made to Andria Ford.
**6**	Payment for expert testimony	☒ **None**	
**7**	Support for attending meetings and/or travel	☒ **None**	
**8**	Patents planned, issued or pending	☒ **None**	
**9**	Participation on a Data Safety Monitoring Board or Advisory Board	☒ **None**	
**10**	Leadership or fiduciary role in other board,	☒ **None**	

		Name all entities with whom you have this relationship or indicate none (add rows as needed)	Specifications/Comments (e.g., if payments were made to you or to your institution)
	society, committee or advocacy group, paid or unpaid		
**11**	Stock or stock options	☒ **None**	
**12**	Receipt of equipment, materials, drugs, medical writing, gifts or other services	☒ **None**	
**13**	Other financial or non-financial interests	☒ **None**	

Please place an "X" next to the following statement to indicate your agreement:

☐ I certify that I have answered every question and have not altered the wording of any of the questions on this form.

FOR US-BASED AUTHORS ONLY>>> OPEN PAYMENTS

For US-based authors (i.e., those with a US National Provider Identifier [NPI]), it is now required they look up their Open Payments record (past 2 years only) to confirm that it matches what is reported on the ICMJE Form. If there are discrepancies, the co-author must provide a brief explanation on the ICMJE Form along with the Open Payment URL person record (e.g., https://openpaymentsdata.cms.gov/physician/1316209). To learn more about this new requirement, please refer to Van Gelder RN and Siegfried CJ. ROI, COI, and the Ethical Obligations of Journals. Ophthalmology. 2022; 129: 602-604.

**Open Payments URL:**	https://openpaymentsdata.cms.gov/physician/599331
**Match Disclosure Form?**	YES
**If no, please briefly explain discrepancy:**	Click or tap here to enter text.

ICMJE FORM

Date: 2/14/2024

Your Name: Rajendra Apte

Manuscript Title: Crizanlizumab for Retinal Vasculopathy with Cerebral Leukoencephalopathy in a Phase 2 Clinical Study

US-based Author (if yes, you must fill out Open Payment section below): YES

Manuscript Number (if known):

Ophthalmology complies with the position of the International Committee of Medical Journal Editors (ICMJE) on "Conflict of Interest." Conflict of interest for authors is defined as "financial and other conflicts of interest that might bias their work."

In the interest of transparency, we ask you to disclose all relationships/activities/interests listed below that are related to the content of your manuscript. "Related" means any relation with for-profit or not-for-profit third parties whose interests may be affected by the content of the manuscript. Disclosure represents a commitment to transparency and does not necessarily indicate a bias. If you are in doubt about whether to list a relationship/activity/interest, it is preferable that you do so.

The author's relationships/activities/interests should be defined broadly. For example, if your manuscript pertains to the epidemiology of hypertension, you should declare all relationships with manufacturers of antihypertensive medication, even if that medication is not mentioned in the manuscript.

In item #1 below, report all support for the work reported in this manuscript without time limit. For all other items, the time frame for disclosure is the past 36 months.

		**Name all entities with whom you have this relationship or indicate none (add rows as needed)**	**Specifications/Comments (e.g., if payments were made to you or to your institution)**
**Time frame: Since the initial planning of the work**			
1	All support for the present manuscript (e.g., funding, provision of study materials, medical writing, article processing charges, etc.) **No time limit for this item.**	☐ None	
		Clayco Foundation	Washington University
		Research to Prevent Blindness	Washington University
		Jeffrey T. Fort Innovation Fund	Washington University
		Siteman Retina Research Fund	Washington University
**Time frame: past 36 months**			
**2**	Grants or contracts from any entity (if not indicated in item #1 above).	☒ **None**	

		Name all entities with whom you have this relationship or indicate none (add rows as needed)	Specifications/Comments (e.g., if payments were made to you or to your institution)
**3**	Royalties or licenses	☒ **None**	
**4**	Consulting fees	☒ **None**	
		Roche	Personal Compensation
**5**	Payment or honoraria for lectures, presentations, speakers bureaus, manuscript writing or educational events	☒ **None**	
**6**	Payment for expert testimony	☒ **None**	
**7**	Support for attending meetings and/or travel	☒ **None**	
**8**	Patents planned, issued or pending	☐ **None**	
**9**	Participation on a Data Safety Monitoring Board or Advisory Board	☒ **None**	
**10**	Leadership or fiduciary role in other board,	☒ **None**	

		Name all entities with whom you have this relationship or indicate none (add rows as needed)	Specifications/Comments (e.g., if payments were made to you or to your institution)
	society, committee or advocacy group, paid or unpaid		
11	Stock or stock options	☒ **None**	
12	Receipt of equipment, materials, drugs, medical writing, gifts or other services	☒ **None**	
13	Other financial or non-financial interests	☒ **None**	

Please place an "X" next to the following statement to indicate your agreement:

☒ I certify that I have answered every question and have not altered the wording of any of the questions on this form.

FOR US-BASED AUTHORS ONLY>>> OPEN PAYMENTS

For US-based authors (i.e., those with a US National Provider Identifier [NPI]), it is now required they look up their Open Payments record (past 2 years only) to confirm that it matches what is reported on the ICMJE Form. If there are discrepancies, the co-author must provide a brief explanation on the ICMJE Form along with the Open Payment URL person record (e.g., https://openpaymentsdata.cms.gov/physician/1316209). To learn more about this new requirement, please refer to Van Gelder RN and Siegfried CJ. ROI, COI, and the Ethical Obligations of Journals. Ophthalmology. 2022; 129: 602-604.

**Open Payments URL:**	https://openpaymentsdata.cms.gov/physician/159619
**Match Disclosure Form?**	NO
**If no, please briefly explain discrepancy:**	Some completely unrelated disclosures on the url link
